# A mouse model for inducible overexpression of *Prdm14* results in rapid-onset and highly penetrant T-cell acute lymphoblastic leukemia (T-ALL)

**DOI:** 10.1242/dmm.012575

**Published:** 2013-09-05

**Authors:** Brandi L. Carofino, Bernard Ayanga, Monica J. Justice

**Affiliations:** 1 Interdepartmental Program in Translational Biology and Molecular Medicine, Baylor College of Medicine, One Baylor Plaza, Houston, TX 77030, USA; 2 Department of Molecular and Human Genetics, Baylor College of Medicine, One Baylor Plaza, Houston, TX 77030, USA

## Abstract

PRDM14 functions in embryonic stem cell (ESC) maintenance to promote the expression of pluripotency-associated genes while suppressing differentiation genes. Expression of *PRDM14* is tightly regulated and typically limited to ESCs and primordial germ cells; however, aberrant expression is associated with tumor initiation in a wide variety of human cancers, including breast cancer and leukemia. Here, we describe the generation of a Cre-recombinase-inducible mouse model for the spatial and temporal control of *Prdm14* misexpression [ROSA26 floxed-stop *Prdm14* (R26PR)]. When R26PR is mated to either of two Cre lines, Mx1-cre or MMTV-cre, mice develop early-onset T-cell acute lymphoblastic leukemia (T-ALL) with median overall survival of 41 and 64 days for R26PR;Mx1-cre and R26PR;MMTV-cre, respectively. T-ALL is characterized by the accumulation of immature single-positive CD8 cells and their widespread infiltration. Leukemia is preceded by a dramatic expansion of cells resembling hematopoietic stem cells and lymphoid-committed progenitors prior to disease onset, accompanied by a blockage in B-cell differentiation at the early pro-B stage. Rapid-onset PRDM14-induced T-ALL requires factors that are present in stem and progenitor cells: R26PR;dLck-cre animals, which express *Prdm14* starting at the double-positive stage of thymocyte development, do not develop disease. PRDM14-induced leukemic cells contain high levels of activated NOTCH1 and downstream NOTCH1 targets, including MYC and HES1, and are sensitive to pharmacological inhibition of NOTCH1 with the γ-secretase inhibitor DAPT. Greater than 50% of human T-ALLs harbor activating mutations in *NOTCH1*; thus, our model carries clinically relevant molecular aberrations. The penetrance, short latency and involvement of the NOTCH1 pathway will make this hematopoietic R26PR mouse model ideal for future studies on disease initiation, relapse and novel therapeutic drug combinations. Furthermore, breeding R26PR to additional Cre lines will allow for the continued development of novel cancer models.

## INTRODUCTION

Cancer is a disease impacting individuals worldwide, with 12 million new diagnoses made in 2008 and a projected 27 million diagnoses expected in the year 2030 (International Agency for Research on Cancer and World Health Organization, 2008). Consequently, research efforts have focused on reducing cancer mortality through the elucidation of the molecular pathways that drive and sustain tumorigenesis after treatment. Drug resistance and cancer relapse are attributed to the persistence of ‘cancer initiating cells’ (CICs), which share many features with normal tissue-resident stem cells, including functional capabilities such as self-renewal, which supports long-term tumor growth, and asymmetric division, which gives rise to the bulk population of heterogeneous, more differentiated cancer cells (Dalerba et al., 2007). As such, stem and progenitor cells are the likely targets for oncogenic insults that lead to transformation and subsequent conversion to CICs. Mutations that promote genomic instability, such as inactivation of DNA maintenance and/or repair factors, often occur early in the multistep process of tumorigenesis. Ongoing genomic instability in cells such as CICs can subsequently allow for outgrowth of unique subclones, which acquire additional driver mutations that endow them with a selective growth advantage (Hanahan and Weinberg, 2011). Furthermore, CICs are believed to be resistant to many conventional cancer treatments, and are consequently responsible for indolent and relapsed disease. Thus, identifying factors that drive CIC growth and evolution, as well as developing therapeutics for targeted eradication of CICs, will be crucial for ensuring a sustained decline in cancer mortality rates.

Aberrant expression of genes that support normal stem cell function might lead to stem cell pool expansion and subsequent formation of CICs. *PRDM14* [PRDI-BF1 (positive regulatory domain I-binding factor 1) and RIZ (retinoblastoma interacting zinc finger) homology domain containing 14] is expressed exclusively in pluripotent cell types, including both mouse and human embryonic stem cells (ESCs) and murine primordial germ cells (PGCs), where it functions as a scaffold to recruit chromatin remodeling or transcription factors to DNA regulatory elements or as a putative histone methyltransferase (Hohenauer and Moore, 2012). In ESCs, PRDM14 supports the maintenance of self-renewal by promoting expression of stem cell markers while also repressing differentiation factors (Chia et al., 2010; Ma et al., 2011; Tsuneyoshi et al., 2008). PRDM14 also facilitates the induction of pluripotency in cells that lack this potential, as demonstrated in epiblast stem cell (EpiSC)-to-ESC reversion and PGC specification, where it orchestrates events such as activation of pluripotency gene expression, global epigenetic reprogramming and X chromosome reactivation (Gillich et al., 2012; Yamaji et al., 2008). Recently, PRDM14 has been shown to downregulate genes through recruitment of polycomb repressive complex 2 (PRC2), and repress *Dnmt3a* and *Dnmt3b* to induce loss of DNA methylation (Chan et al., 2013; Grabole et al., 2013; Leitch et al., 2013).

**RESOURCE IMPACT**

**Background**

Acute lymphoblastic leukemia (ALL) is the most common childhood malignancy and is the leading cause of cancer-related death among children worldwide. Cancer-initiating cells (CICs), which are cells with stem-cell-like properties that give rise to heterogeneous, more differentiated cancer cells, are hypothesized to be the source of indolent and relapsed disease. These cells might reside in a protective niche and cycle slowly, and are not eradicated by traditional chemotherapeutics that primarily target rapidly dividing cells. Thus, there is a definitive need in the field to identify, characterize and develop novel therapeutics that directly target CICs while sparing the normal stem cell compartment. *PRDM14*, a potency gene implicated in a wide variety of cancers, has been proposed to be a driver of CICs. An animal model that allows targeted, inducible expression of this gene would be useful for studying the mechanisms of ALL initiation and progression *in vivo*.

**Results**

The authors developed a novel Cre-recombinase-inducible mouse model that facilitates spatial and temporal regulation of *Prdm14* misexpression. To verify the utility of the model, they overexpressed *Prdm14* in hematopoietic progenitor cells. This resulted in rapid development of ALL in the T-cell population (T-ALL) of all the mice tested, and the mice died of the disease within 2 months. Overexpression of *Prdm14* in differentiated T-cells did not induce disease. Finally, the authors confirmed that the PRDM14-induced mouse tumors share molecular features with human T-ALL, including the expression of high levels of activated NOTCH1, which is mutated in more than 50% of human T-ALL cases.

**Implications and future directions**

In the PRDM14 leukemia mouse model described here, every animal develops T-ALL and disease progression is extremely rapid. This does not hold true for many of the leukemia mouse models that are currently available. Thus, this model provides a powerful tool for future studies aimed at evaluating the efficacy of novel cancer drugs or combinations of existing therapeutics. Because *PRDM14* is not expressed beyond embryogenesis, it could represent an ideal druggable target in new anti-cancer therapies. The inducible model is also extremely flexible, and can be used to overexpress *Prdm14* in other tissues such as the mammary gland to model solid tumors such as breast cancer. Future studies will elucidate the molecular changes that occur following *Prdm14* overexpression and shed light on how these changes contribute to cancer development and progression.

Based on its normal function, misexpression of *PRDM14* beyond the milieu of germ cell development could promote cellular de-differentiation, hyperproliferation and transformation. Indeed, *PRDM14* overexpression has been detected in a variety of human cancer types, including non-small cell lung cancer, T-cell acute lymphoblastic leukemia (T-ALL), high hyperdiploid pre-B-ALL, and breast cancer (Liu et al., 2010; Dettman et al., 2011; Hu et al., 2005; Moelans et al., 2010; Nishikawa et al., 2007). *PRDM14* overexpression is often correlated with genomic amplification (Forozan et al., 2000), and amplification and/or overexpression is significantly associated with high mitotic index, high histological grade and HER2 positivity of invasive breast cancer specimens (Moelans et al., 2010), as well as chemoresistance to cisplatin, etoposide, docetaxel and doxorubicin in cultured breast cancer cell lines (Nishikawa et al., 2007). These high-risk features of PRDM14-expressing cancers reinforce the significance of determining its molecular function in tumorigenesis.

As a potential multi-cancer oncogene, a single mouse model that allows for inducible expression of *Prdm14* in specific cell types is desirable. Whereas classical transgenic approaches are confounded by random integration and position effects (Frese and Tuveson, 2007), knock-in approaches targeting a known locus, such as the well-characterized and widely used ROSA26 locus, has many advantages: ROSA26 is ubiquitously expressed, disrupting the endogenous transcripts has no apparent detrimental phenotype (Zambrowicz et al., 1997) and targeted oncogenes can be conditionally overexpressed in Cre-expressing tissues by placing a *loxP-*STOP-*loxP* ‘floxed STOP (FS)’ (Kühn and Torres, 2002) element upstream of the gene of interest. A large number of inducible and tissue-specific Cre recombinase transgenic lines are readily available (Nagy et al., 2009), making this system a tractable approach for modeling multiple cancer types. Here, we describe the development of a novel mouse model for inducible expression of *Prdm14* by targeting FS-*Prdm14* to the ROSA26 locus and breeding to multiple Cre-recombinase transgenic lines. Not only does this model work efficiently, but lymphoblastic leukemia develops with a very short latency, making this model a robust method for determining the molecular function of PRDM14 and its role in tumorigenesis. The rapid onset and full penetrance of tumors will allow for the development of strategies to target CICs in this model.

## RESULTS

### Generation of a ROSA26-FS-*Prdm14* vector and ESC targeting

**Fig. 1.**
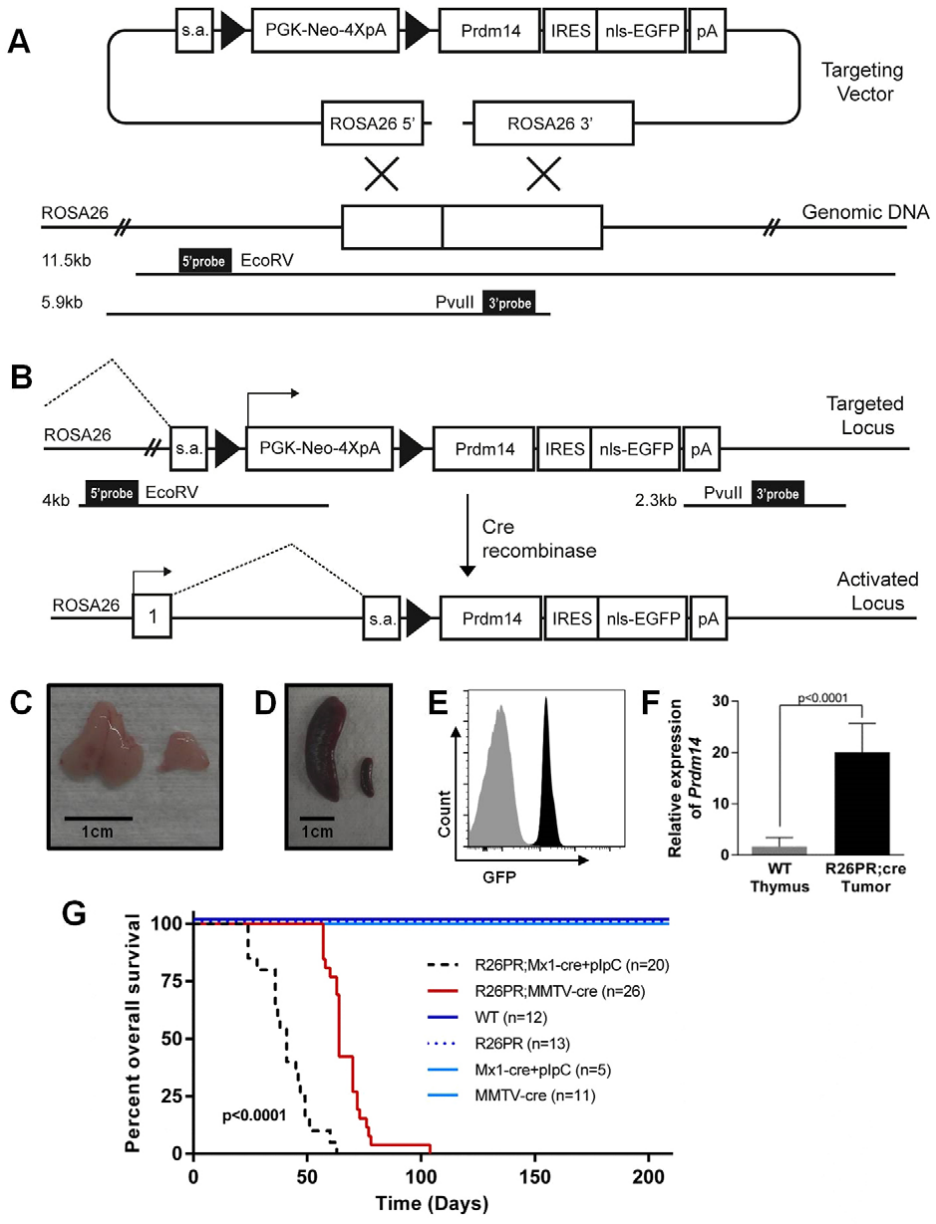
**Activation of R26PR results in the rapid development of leukemia.** (A) ROSA26 targeting vector, which includes: 5′ and 3′ ROSA26 homology arms, a splice acceptor (s.a.), *loxP* sequences (black triangles), a PGK-Neo-4×poly(A) stop sequence, and cDNA for *Prdm14* and an IRES-nls-EGFP fluorescent marker (R26PR). Southern blot probes and enzymes are indicated at the bottom. (B) Schematic of the targeted locus during Cre-mediated floxed stop excision. (C,D) Thymus (C) and spleen (D) of diseased animal (left) compared with WT (right). (E) GFP histogram for diseased (black) and WT (gray) thymus. (F) qRT-PCR for *Prdm14* mRNA in thymi of R26PR;MMTV-cre animals compared with WT, R26PR-only or Cre-only controls. (G) Overall survival of R26PR;Mx1-cre+pIpC (*n*=20), R26PR;MMTV-cre (*n*=26), WT (*n*=12), R26PR only (*n*=13), Mx1-cre+pIpC only (*n*=5) and MMTV-cre only (*n*=11) controls.

We developed a construct targeting the conditional FS-*Prdm14* cassette to the ROSA26 locus (R26PR; Fig. 1A) as previously described (Hohenstein et al., 2008). The presence of the upstream 4×-polyadenylation [4×-poly(A)] sequence terminates transcription and prevents expression of the downstream elements, including *Prdm14.* Upon exposure to Cre recombinase, sequences between *loxP* sites, including the 4×-poly(A) sequence, are excised. Transcription of *Prdm14* is then driven from the constitutively active ROSA26 promoter, leading to expression of the transgene and downstream EGFP reporter only in cells expressing Cre (Fig. 1B). Southern blotting confirmed appropriate genomic targeting (supplementary material Fig. S1A). Chimeric animals were generated by injecting R26PR ESCs [derived from the C57BL/6N line JM8A3 (described in Pettitt et al., 2009)] into C57BL/6-*Tyr^c-Brd^* (albino) blastocysts. Germline transmission testing of chimeric males (supplementary material Fig. S1B,C) produced a total of 118 pups, 35 of which had black or agouti coats (supplementary material Fig. S1D). The breeding strategy described here allows for rapid identification of germline transmission at birth by the presence of visible dark eyes compared with the light-colored eyes of albino pups (supplementary material Fig. S1E). Animals carrying the targeted allele were backcrossed to the C57BL/6J line to generate a stable colony carrying the R26PR allele.

### Hematopoietic expression of *Prdm14* results in rapid-onset leukemia

We previously demonstrated that *Prdm14* is a potent leukemia oncogene, which can cause B-, T- or mixed-lineage lymphoblastic leukemia when expressed in stem-cell-enriched bone marrow via retroviral transduction, albeit with incomplete penetrance and a long latency of 12–52 weeks (Dettman et al., 2011). To confirm the utility of the R26PR mouse model, we mated R26PR mice to the hematopoietic-specific Cre-recombinase-expressing line Tg(Mx1-cre)1Cgn/J (hereafter called Mx1-cre) to determine whether deletion of the FS cassette and subsequent expression of *Prdm14* in R26PR animals would also result in leukemia. Mx1-cre is activated by injecting synthetic double-stranded RNA [polyinosinic-polycytidylic acid (pIpC)] to induce an interferon response, which drives expression from the Mx1 promoter (Kühn et al., 1995). Additionally, to expand our model to a novel system, we mated R26PR to Tg(MMTV-cre)4Mam/J (Line D; hereafter called MMTV-cre), in which Cre expression is driven by the mouse mammary tumor virus (MMTV) long terminal repeat (LTR), which is expressed in the mammary gland with previous reports of off-target expression in skin, salivary gland, B-cells, T-cells, megakaryocytes and erythroid cells (Wagner et al., 2001). Although PRDM14 has been implicated in human breast cancer, a mouse model misexpressing *Prdm14* in the mammary gland has not been developed. Surprisingly, both the hematopoietic R26PR;Mx1-cre and mammary R26PR;MMTV-cre lines very rapidly developed and succumbed to acute leukemia. Diseased animals had extremely distended abdomens, palpable enlarged lymph nodes, labored breathing and lethargy. Animals also had enlarged thymi (Fig. 1C), livers, kidneys and spleens (Fig. 1D). Flow cytometry for GFP-expressing cells (Fig. 1E) and qRT-PCR on R26PR;MMTV-cre (Fig. 1F) and R26PR;Mx1-cre tumors (data not shown) (*t*-test, *P*<0.0001) confirmed expression of *Prdm14* within tumors. Overall survival was significantly reduced (log-rank test, *P*<0.0001), with median survival of 41 days post-pIpC injection and 64 days of life for the R26PR;Mx1-cre and R26PR;MMTV-cre lines, respectively (Fig. 1G). Control animals carrying only R26PR, Mx1-cre (with pIpC injection), MMTV-cre or no transgenic/targeted alleles [wild type (WT)] did not develop cancer or any other disease after 6 months of aging. A PCR assay validated the Cre-mediated excision of the FS cassette (supplementary material Fig. S2A), which occurred only in tumors. The leukemia phenotype was completely penetrant; all animals developed disease in both R26PR;cre lines.

### Systemic features of R26PR;cre-induced leukemia

**Fig. 2.**
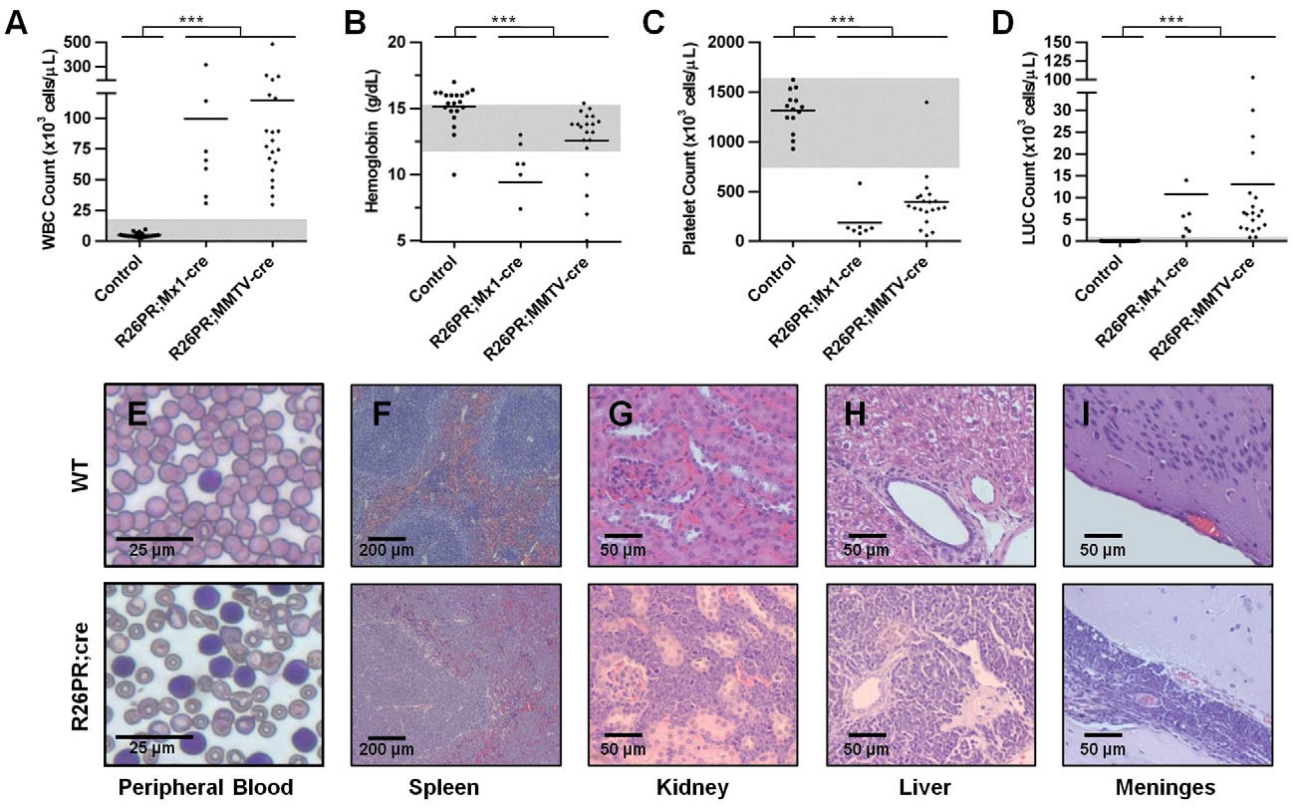
**Leukemic animals exhibit lymphocytosis, organ infiltration, anemia and thrombocytopenia.** (A) White blood cell (WBC), (B) hemoglobin, (C) mean platelet and (D) large unstained cells (LUC) counts in peripheral blood of control (WT, MMTV-cre-only and R26PR-only) animals at 6 months compared with moribund animals of both R26PR;cre lines. Normal range is indicated by the gray shaded region. Groups were compared using one-way ANOVA with Dunnett’s test for multiple comparisons. ***P<0.001. (E) Representative images of Giemsa-stained blood smears and (F–I) H&E organ histology from WT healthy (top row) and R26PR;cre diseased (bottom row) animals.

Peripheral blood was isolated from moribund animals prior to sacrifice and from control animals at 6 months of age. The white blood cell count was significantly higher in R26PR;Mx1-cre (*n*=7, mean 99.4×10^3^ cells/μl) and R26PR;MMTV-cre (*n*=20, mean 114.4×10^3^ cells/μl) animals than in controls (*n*=20, mean 5×10^3^ cells/μl) (Fig. 2A). Diseased animals were also slightly anemic (hemoglobin mean 9.4 and 12.5 g/dl versus 15.2 g/dl, respectively; Fig. 2B) and severely thrombocytopenic (platelet count mean 184×10^3^ cells/μl and 395×10^3^ cells/μl versus 1312×10^3^ cells/μl, respectively; Fig. 2C). Finally, a preponderance of large unstained cells, or abnormal blasts, were present in diseased animals (mean 10.8×10^3^ cells/μl and 13×10^3^ cells/μl) but were primarily absent in controls (mean 0.035×10^3^ cells/μl) (Fig. 2D). A monomorphic population of enlarged white blood cells with a high nuclear:chromatin ratio, consistent with leukemic blasts, was present on peripheral blood smears (Fig. 2E). In addition to abnormal blood counts, histological analysis revealed widespread dissemination of lymphoblasts in diseased animals. Healthy splenic tissue had clear separation between white and red pulp, whereas this was disrupted by infiltrating lymphoblasts in diseased animals (Fig. 2F). Furthermore, lymphoid infiltration was observed in the interstitial and perivascular space of the kidney (Fig. 2G), as perivascular cuffs in the liver (Fig. 2H), within the meninges surrounding the brain (Fig. 2I), and in the thymus, stomach and intestine (data not shown).

### R26PR;cre-induced acute leukemia is T-cell in origin (T-ALL)

**Fig. 3.**
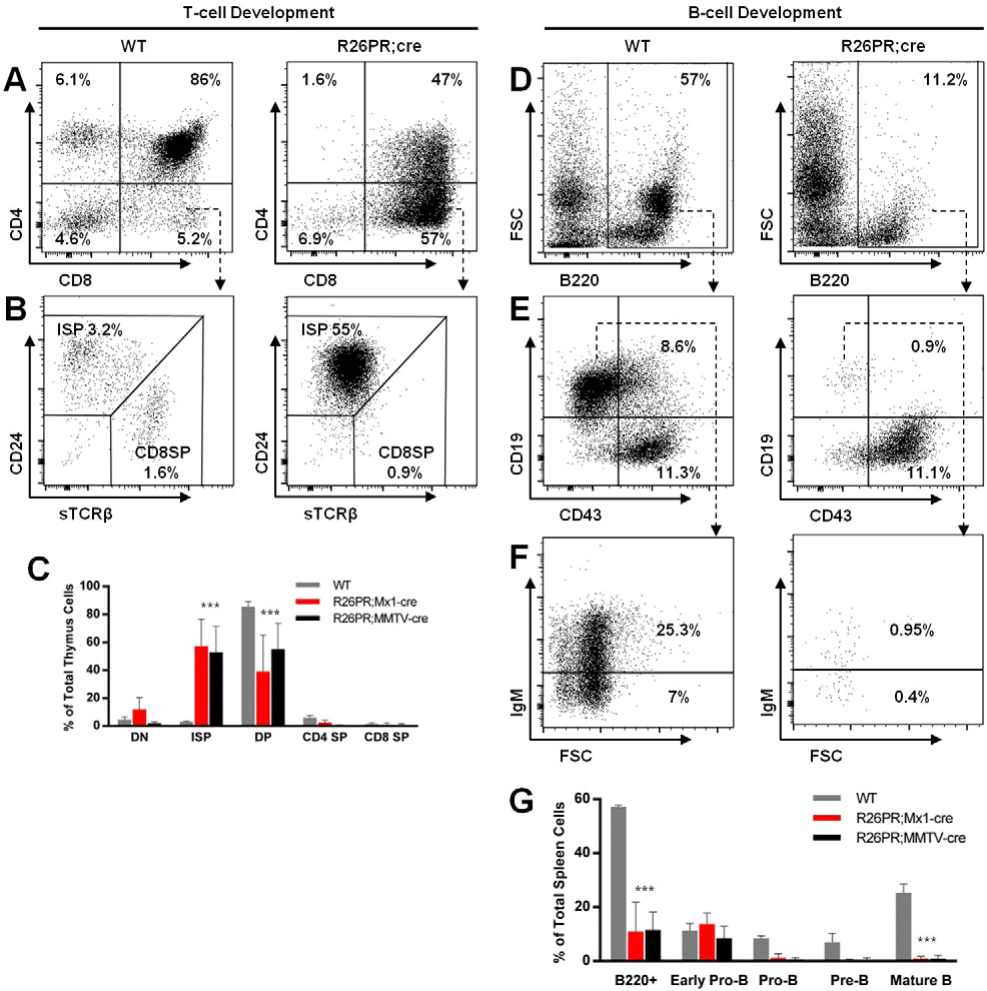
**Leukemia involves the accumulation of ISP CD8^+^T-cells and blockages in B-cell development.** (A) Thymic T-cell distribution based on CD4 and CD8 expression. (B) CD8^+^ cells are separated according to CD24 and surface TCRβ expression. (C) Summary of T-cell development data, shown as a percent of total thymus cells, for WT (*n*=6), R26PR;Mx1-cre+pIpC (*n*=6) and R26PR;MMTV-cre (*n*=8). (D) Total number of splenic B220^+^ B-cells. FSC, forward scatter. (E) B220^+^ gated cells are separated according to CD19 and CD43 expression. Cells are blocked at the early-pro-B stage in R26PR;cre animals and had reduced progression to the pro-B stage compared with controls. (F) CD19^+^CD43^−^ gated cells are separated according to surface IgM expression for pre-B and mature B-cell discrimination. (G) Summary of B-cell development data, shown as a percent of total spleen cells, for WT (*n*=4), R26PR;Mx1-cre+pIpC (*n*=6) and R26PR;MMTV-cre (*n*=8). Groups were compared using one-way ANOVA with Dunnett’s test for multiple comparisons. ***P<0.001. Representative flow plots show averages of R26PR;Mx1-cre and R26PR;MMTV-cre values in each panel.

We performed flow cytometric analysis to characterize the cellular distribution of the R26PR;Mx1-cre and R26PR;MMTV-cre leukemias. Phenotypes of both lines were indistinguishable, and representative panels and combined averages are shown in Fig. 3. Analysis of T-cell development in the thymus showed that, whereas most thymic T-cells were CD4^+^CD8^+^ double-positive (DP) in WT animals (86%, versus 47% in R26PR;cre), most were CD8^+^ in leukemic animals (57%, versus 5.2% in WT) (Fig. 3A). The CD8^+^ population contained primarily immature single-positive (ISP; CD24^+^TCRβ^lo^) CD8^+^ cells (mean 3.2% in WT versus 55% in R26PR;cre) (Fig. 3B). The number of ISP cells was significantly higher and DP cells significantly lower for R26PR;cre compared with WT (Fig. 3C). The same leukemic T-cell population was present in other tissues, including spleen and bone marrow (data not shown).

Analysis of splenic B-cell development found fewer total B220^+^ B-cells (Fig. 3D) (11.2% in R26PR;cre versus 57% in WT), and nearly all were arrested at the early pro-B (CD19^−^CD43^+^) stage of development in R26PR;cre animals (11.1%), with very limited numbers of pro-B (0.9%), pre-B (0.4%) or mature B-cells (0.95%) (Fig. 3E,F). Any cells at the pro-B stage or beyond in R26PR;cre animals were GFP^−^, which indicates that they were derived from progenitors not expressing the R26PR construct (data not shown). B-cell data are summarized in Fig. 3G.

Southern blotting and PCR analysis of B-cell and T-cell receptor (BCR and TCR, respectively) locus rearrangements within tumors and control tissues confirmed cell-of-origin and clonality of disease. Tumor DNA contained the germline configuration for BCR loci (IgH and IgK), but showed rearrangement of TCR loci (Jβ1 and Jβ2), confirming T-cell origin of disease (supplementary material Fig. S3A). Either single or multiple dominant TCR configurations were found within a single tumor, suggesting either monoclonal or early polyclonal expansion during disease development, respectively. Additionally, PCR analysis found the same monoclonal dominant clone in both the spleen and thymus, indicating that peripheral disease was derived from the original tumor (supplementary material Fig. S3B).

### MMTV-cre is expressed in hematopoietic stem cells, which are also expanded prior to disease onset

**Fig. 4.**
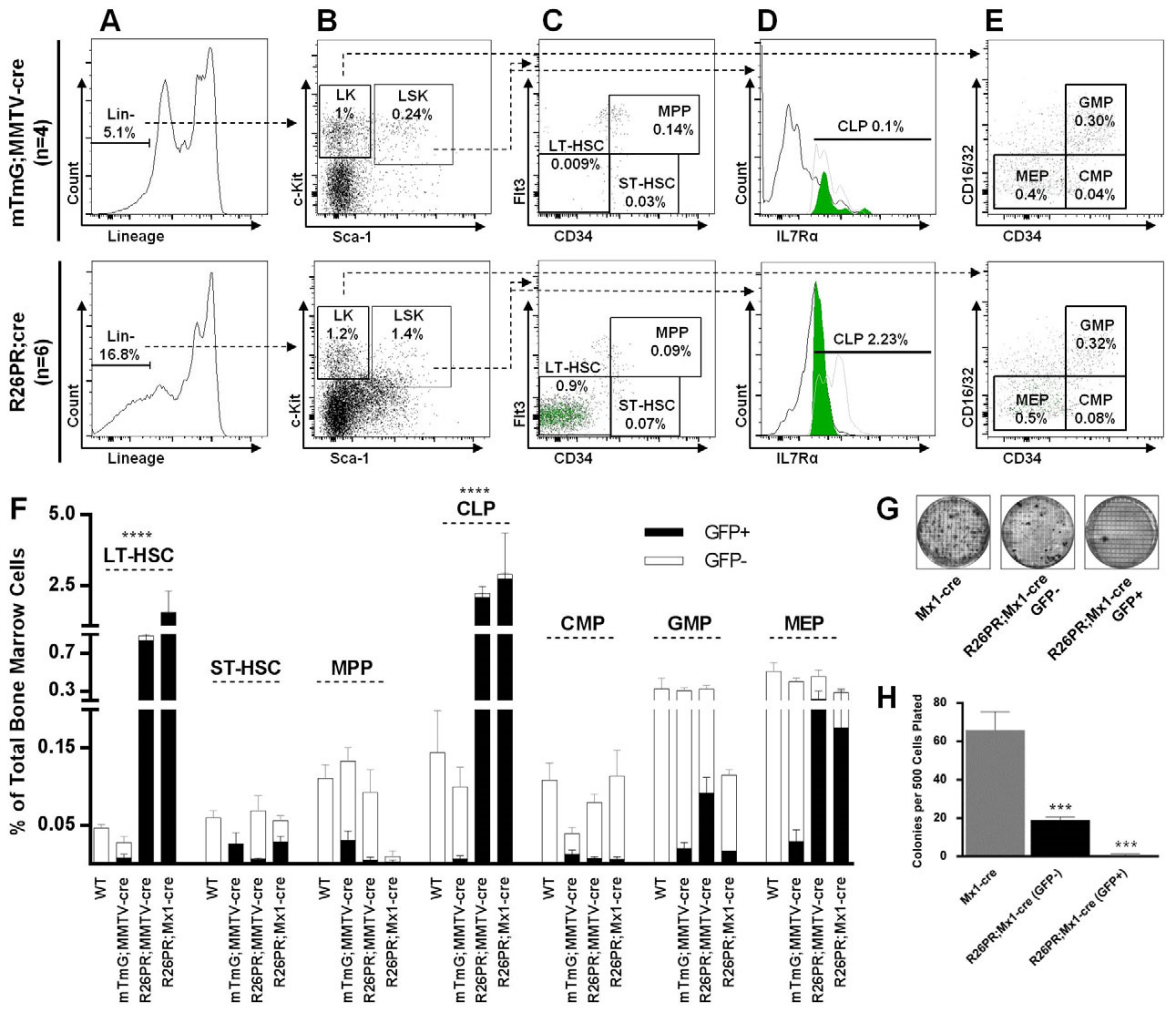
**LT-HSCs are expanded and preferentially give rise to CLPs prior to disease onset.** Bone marrow was analyzed 2 weeks prior to disease onset in WT (*n*=3), mTmG;MMTV-cre (GFP Cre reporter, *n*=4), R26PR;MMTV-cre (*n*=6) and R26PR;Mx1-cre (*n*=4) animals. (A) Total lineage-negative (Lin^−^) cells are gated to next panel. (B) Lin^−^ cells are separated according to Kit and Sca-1 status and gated into LK (Lin^−^c-Kit^+^) and LSK (Lin^−^c-Kit^+^Sca-1^+^) fractions. (C) LSK gated cells are plotted based on Flt3 and CD34 expression to evaluate percentages of LT-HSCs, ST-HSCs and MPPs. GFP^+^ cells are overlaid as green dots on the plot. (D) LSK gated cells are evaluated for IL7Rα expression. The LSK^+^IL7Rα histogram (black line) is overlaid with a GFP^+^ (filled green) and GFP^−^ (gray line) CLP histogram. (E) LK gated cells are plotted based on CD16/32 and CD34 expression to evaluate percentages of CMPs, GMPs and MEPs. (F) Summary of preleukemic cells for all lines according to GFP status (GFP^+^ filled black, GFP^−^ white). One-way ANOVA with Dunnett’s test for multiple comparisons was used to compare the GFP^+^ mTmG;MMTV-cre population with the GFP^+^ R26PR;MMTV-cre and R26PR;Mx1-cre populations. ****P<0.0001. (G) Representative images of MethoCult assay plates to assess myeloid developmental potential, with 500 Lin^−^Sca1^+^cKit^+^CD150^+^ (GFP^+^ and GFP^−^) cells per indicated replicate plate, with mean counts summarized in H. Groups were compared using one-way ANOVA with Dunnett’s test for multiple comparisons. ***P<0.001.

MMTV-cre is reported to be expressed in lymphoid and myeloid cells (Wagner et al., 2001; Robinson and Hennighausen, 2011); however, the basis for and extent of hematopoietic expression is poorly described. To understand the basis for T-ALL development in the R26PR;MMTV-cre line and to characterize the cellular changes taking place prior to disease development, we mated MMTV-cre mice to a Cre reporter line that permanently tags Cre-expressing cells with GFP (mTmG) (Muzumdar et al., 2007) and compared the GFP^+^ and GFP^−^ hematopoietic progenitor populations in bone marrow of WT, mTmG;MMTV-cre, R26PR;Mx1-cre and R26PR;MMTV-cre animals approximately 2 weeks prior to disease onset (Fig. 4A–F). In the mTmG;MMTV-cre line, GFP^+^ cells were present within the long-term hematopoietic stem cell (LT-HSC) population (∼30% of the total LT-HSC population) and in all subsequent differentiated lineages (Fig. 4F), explaining the basis for both lymphoid and myeloid expression of MMTV-cre-activated alleles.

The LT-HSC population was massively expanded in both R26PR;cre lines, making up ∼1% of the total bone marrow (versus 0.009% in mTmG;MMTV-cre), 100-times higher than controls (Fig. 4C,F), with no statistically significant changes in either the total or ratio of GFP^+^:GFP^−^ within the short-term HSC, multi-potent progenitor (MPP), common myeloid progenitor (CMP), granulocyte-monocyte progenitor (GMP) or megakaryocyteerythroid progenitor (MEP) populations (Fig. 4E,F). However, the common lymphoid progenitor (CLP) compartment was expanded over 20-fold (2.23 versus 0.1%) in the R26PR;cre lines compared with controls (Fig. 4D,F). The expanded LT-HSC and CLP populations consisted nearly exclusively of GFP^+^ cells. Therefore, a dramatic expansion of both early hematopoietic stem cells and lymphoid-committed progenitors occurs in the bone marrow prior to disease onset in the R26PR;cre lines.

Because we observed an expansion of lymphoid but not myeloid progenitors, we performed a colony-forming assay, in which methylcellulose is supplemented with cytokines that support myeloid progenitor outgrowth, to test the multi-lineage differentiation potential of the LT-HSC population. Sorted HSCs were plated, and R26PR;Mx1-cre cells were further separated according to GFP status (Fig. 4G). Significantly fewer colonies grew from both the R26PR;Mx1-cre-GFP^+^ (mean 0.6 colonies/500 cells plated) and R26PR;Mx1-cre-GFP^−^ (19 colonies) populations as compared with Mx1-cre (65.8 colonies) (Fig. 4H). The reduced myeloid potential of the R26PR;Mx1-cre-GFP^−^ population might be due to its contamination by GFP^+^ cells or misidentification of cells because of incomplete GFP translation from the internal ribosome entry site (IRES). Despite this, our data still clearly show that *Prdm14*-expressing LT-HSCs preferentially contribute to lymphoid but not myeloid lineages, and therefore do not have multi-lineage potential. Consequently, these cells should more accurately be referred to as cells with an ‘LT-HSC immunophenotype’ or ‘LTHSC-like cells’.

### *Prdm14* does not induce T-ALL when overexpressed in differentiated T-cells

**Fig. 5.**
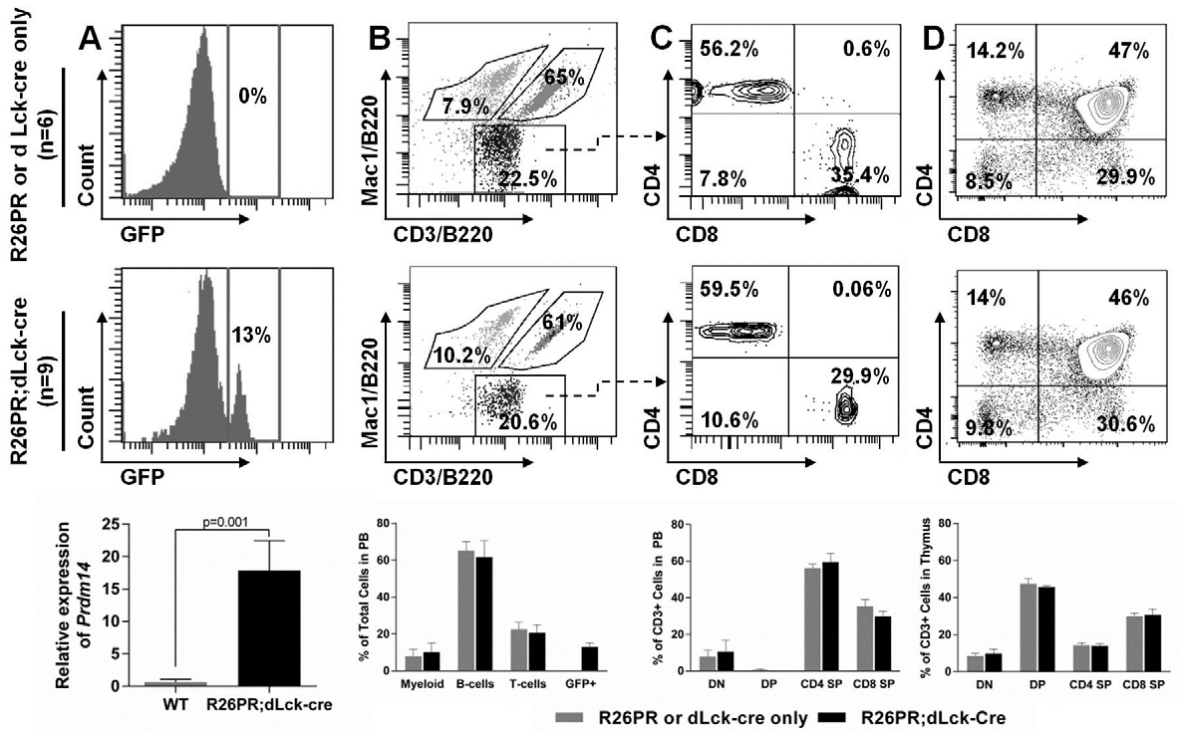
**Cellular distribution in the blood and thymus is not altered in R26PR;dLck-cre mice.** Representative plots for control animals (R26PR and dLck-cre only, *n*=6) and R26PR;dLck-cre (*n*=9) animals are shown in the top and middle panels, respectively, and summary graphs are shown in the bottom panel. (A) Analysis of GFP^+^ cells in peripheral blood and thymic expression of *Prdm14*. (B) Distribution of myeloid (Mac1^+^B220^−^), B-cells (B220^+^) and T-cells (CD3^+^) in the peripheral blood. (C) CD3^+^ cells are subdivided according to CD4 and CD8 markers. (D) Thymic CD3^+^ T-cells are also fractionated based on CD4 and CD8 expression.

We next investigated whether *Prdm14* could induce disease when misexpressed in mature T-cells by allowing differentiated T-cells to acquire self-renewal characteristics, or if it preferentially transforms stem/progenitor cells. The Tg(Lck-cre)3779Nik/J line is driven by the distal promoter of the *Lck* gene (dLck) and drives Cre expression in differentiated T-cells after positive selection during the TCR^hi^ stage of development (Zhang et al., 2005). R26PR;dLck-cre animals did not develop T-ALL; they were indistinguishable from control animals and still healthy at 8 months (*n*=12 animals; data not shown), far beyond the latency observed from the other lines. In spite of their disease-free status, R26PR;dLck-cre animals had detectable GFP^+^ cells in their peripheral blood (Fig. 5A, middle), genomic DNA corresponding to the active R26PR allele in the thymus (supplementary material Fig. S2B) and thymic expression of *Prdm14* (Fig. 5A, bottom), indicating that dLck-cre successfully activated the R26PR allele. In the peripheral blood, both control and R26PR;dLck-cre animals had a similar distribution of cells among the myeloid, B-cell and T-cell lineages (Fig. 5B). The T-cell population in the peripheral blood was split between CD4^+^ and CD8^+^ cells for all animals (Fig. 5C). Additionally, the T-cell distribution in the thymus was indistinguishable between the control and R26PR;dLck-cre groups (Fig. 5D). When R26PR;dLck-cre cells were gated for GFP^+^ cells, an identical immunophenotype was observed (data not shown), further suggesting no perturbation in T-cell development. The normal phenotype of R26PR;dLck-cre animals indicates that PRDM14 more efficiently transforms stem/progenitor cells than it does more differentiated cells.

### High levels of activated NOTCH1 are present in PRDM14-induced tumors

**Fig. 6.**
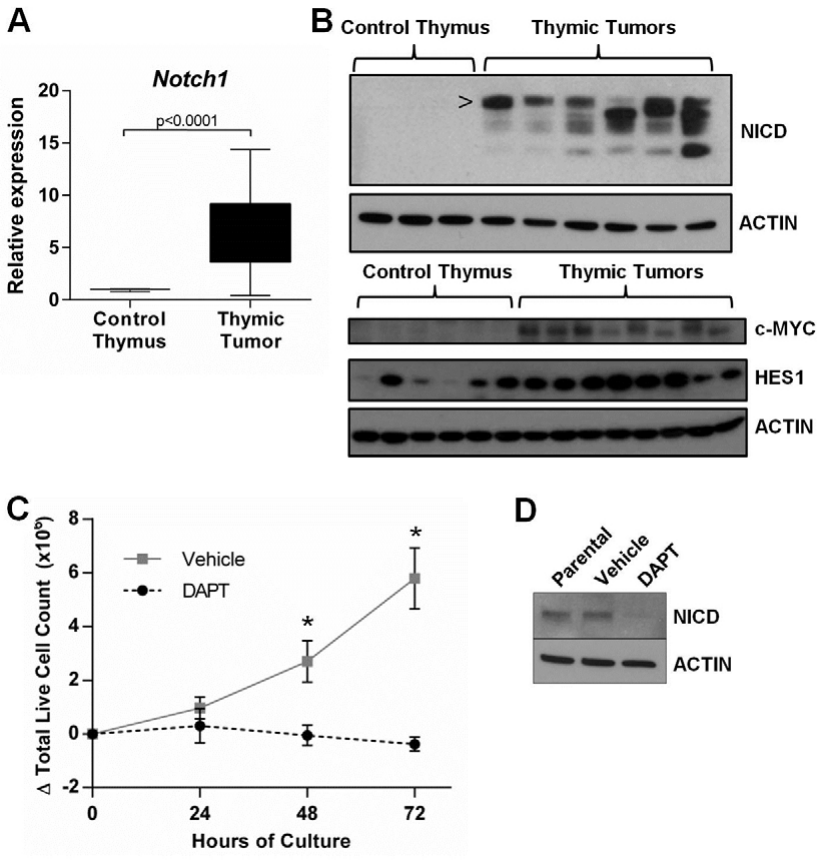
**R26PR;cre tumors express high levels of NICD and are sensitive to pharmacological inhibition of NOTCH1 signaling.** (A) qRT-PCR analysis of *Notch1* transcript levels in control thymic tissue and thymic tumor tissue. (B) Upper panel: western blot analysis of control and thymic tumor tissue. NOTCH1 intracellular domain (NICD) is the activated intracellular portion of NOTCH1 following γ-secretase cleavage. > indicates the NICD (smaller bands are degradation products). Lower panel: western blot analysis for levels of the NOTCH1 targets MYC and HES1. ACTIN was used as a loading control. (C) A cell line derived from R26PR;MMTV-cre tumor cells was cultured in the presence of a γ-secretase inhibitor, DAPT, or DMSO vehicle. Live cells were counted at 24, 48 and 72 hours of culture. (D) Western blot analysis of NICD following DAPT treatment.

NOTCH1 signaling is crucial for normal thymocyte proliferation, differentiation and survival; activating mutations in NOTCH1 occur in more than 50% of human T-ALL cases (Grabher et al., 2006). To determine the role of NOTCH1 in the R26PR;cre murine TALL model, we evaluated *Notch1* mRNA and cleaved/activated NOTCH1 intracellular domain (NICD) levels. Both overall *Notch1* transcript levels and NICD levels were significantly higher in thymic tumors as compared with control thymic tissue (Fig. 6A,B). Several key targets of NOTCH1 include *Myc* and *Hes1*, which signal downstream of NOTCH1 to propagate signals related to cell proliferation, growth and survival. Both MYC and HES1 protein levels were higher in thymic tumors than control thymic tissue (Fig. 6B), indicating that downstream NOTCH1 targets are also activated. To determine the role of NOTCH1 in driving proliferation of R26PR-derived T-ALLs, we cultured a tumor cell line derived from these animals in the presence of the γ-secretase inhibitor N-[N-(3,5-difluorophenacetyl)-L-alanyl]-S-phenylglycine t-butyl ester (DAPT), which inhibits the release of NICD from the cell membrane. Tumor cells cultured in DAPT were unable to proliferate (Fig. 6C), with a concomitant decrease in NICD expression (Fig. 6D) when compared with the parental, untreated cell line and DMSO-vehicle-treated control culture.

## DISCUSSION

The R26PR mouse line described here is a powerful model for the spatial and temporal control of *Prdm14* misexpression, which can be used to investigate the role of PRDM14 in multiple cancer types. Currently, many cancer studies utilize xenograft models, which might not fully recapitulate human disease because they are often derived from ectopically placed cultured cells in immunocompromised mice, and thus lack both the microenvironment and immune cells that play key roles in disease progression (Becher and Holland, 2006; Chew et al., 2012). Leukemia studies frequently make use of transgenic expression, which is limited by founder-to-founder variance, or viral transduction of bone marrow, which is limited by the requirement for successful transduction and engraftment. For instance, we previously pursued a PRDM14 bone marrow transplant model (Dettman et al., 2011), but the approach was limited by variable tumor immunophenotypes (65% were B-cell lineage, 12% T-cell, 19% mixed, 1% CLP and 3% stem-like), incomplete penetrance and long latency (ranging from 12 weeks to 1 year). The variability induced by *ex vivo* manipulation of bone marrow and transduction of mixed lineages made this model difficult to completely characterize. Our new model circumvents the abovementioned issues because it is innately syngeneic and orthotopic without the need for host irradiation or transplant, is activated in consistent subsets of cells using Cre drivers, and results in homogeneous T-ALL with a predictable time of onset. This feature in particular allows us to study tumors prior to their onset, an important consideration for further studies of CICs.

R26PR;cre-induced T-ALLs occur with exceptionally short latency, with median overall survival of 41 days post-pIpC injection and 64 days from birth for the R26PR;Mx1-cre and R26PR;MMTV-cre lines, respectively. For most other T-ALL models described in the literature, disease does not manifest until 6 months to 1 year of age (Aifantis et al., 2008; Chervinsky et al., 1999; Keating et al., 2006; Thoms et al., 2011). Although some bone marrow transplant models have slightly shorter latency of onset than this, occurring around 125–150 days, including an Mx1-cre;*Kras*-LSL-G12D model (Dail et al., 2010) and a *ZMIZ*/mutant *NOTCH1* model (Rakowski et al., 2013), this timeframe is still considerably longer than the R26PR;cre model.

The early development of T-ALL requires *Prdm14* expression in a stem/progenitor compartment, because breeding R26PR to Mx1-cre (+pIpC), which is expressed in hematopoietic stem cells, rapidly induces disease, whereas dLck-cre, which is expressed at late (DP) stages of thymocyte development, did not induce disease by 8 months of age. Others have described increasing disease latency when using Cre drivers expressed in sequentially more differentiated cell types [Vav-cre/HSCs, proximal promoter pLckcre/DN (double negative) thymocytes, and CD4-cre/DP thymocytes] to drive Sleeping Beauty transposon mobilization, with average survival increasing from 11 weeks in the Vav-cre group to 45 weeks in the Lck-cre group and 49 weeks in the CD4-cre group (Berquam-Vrieze et al., 2011). The same phenomenon has been documented for another stem cell self-renewal gene, *Hhex*. When pLck-*Hhex* transgenic mice were aged, only mild perturbations in T-cell development and one case of T-cell lymphoma developed at 13 months (Mack et al., 2002). However, when expressed in hematopoietic precursors using bone marrow transplant models, induction of both thymocyte self-renewal (McCormack et al., 2010) and T-cell lymphoma (George et al., 2003) occurred. Because both PRDM14 and HHEX function in stem cell self-renewal, both might require cooperation with other stem cell factors to efficiently induce disease. Continued aging or genomic insult of R26PR;dLck-cre animals might result in disease development driven by the accumulation of additional driver mutations that cooperate with PRDM14.

The R26PR;MMTV-cre line develops T-ALL that is strikingly similar to the Mx1-cre-driven model. Hematopoietic malignancies have been reported by other groups using MMTV-cre to drive expression of mutant gene isoforms or to delete tumor suppressor genes (Wagner et al., 2000; Wittschieben et al., 2010; Adams et al., 2011), yet the hematopoietic cell types expressing MMTV-cre have not been fully described. Here, we definitively show for the first time that MMTV-cre can excise floxed sequences in LT-HSCs, a genetic event that is propagated downstream to all other hematopoietic cells. This is probably due to the responsiveness of the MMTV-LTR to glucocorticoids (Ross and Solter, 1985), receptors for which are expressed in hematopoietic cells throughout development (Plaut, 1987). Although we intended to develop R26PR;MMTV-cre as a mammary tumor model, animals succumbed to T-ALL prior to completion of mammary gland maturation. Furthermore, fat pad loss, cachexia and lymphoid cell infiltration limit the evaluation of mammary gland development in this model. Therefore, alternative approaches such as transplantation are needed to study *Prdm14* overexpression in the mouse mammary gland.

**Fig. 7.**
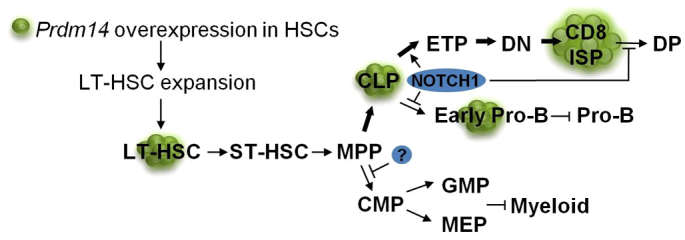
**Model for PRDM14-induced leukemogenesis.** Both the MMTV-cre and Mx1-cre lines drive activation and expression of *Prdm14* in LT-HSCs. This pool of cells expands dramatically (green cells), and is then susceptible to the accumulation of additional driver mutations. *Notch1* activation in these expanded cells (blue oval) promotes lymphoid skewing (accompanied by CLP expansion) and T-cell versus B-cell developmental bias, which manifests as a reduction in total B-cell numbers and arrest at the early pro-B stage of development. These features phenocopy models of NOTCH1 overexpression in HSCs, making *Notch1* activation a probable early event in leukemogenesis. Myeloid development is also inhibited, either by NOTCH1, PRDM14 or another factor. T-cells arrest primarily at the CD8 immature single-positive stage of development, accumulate and infiltrate nearly all organs. Leukemias are completely penetrant and occur rapidly, with overall survival of only 41–64 days for the R26PR;cre lines used in this study.

In the R26PR;cre model, T-ALL is preceded by a massive expansion of LT-HSC-like cells (100-fold) and lymphoid progenitors (20-fold). Furthermore, these LT-HSC-like cells lack multi-lineage potential, as they could not form myeloid progenitor colonies in a methylcellulose assay. Many factors influence HSC self-renewal, proliferation and differentiation, but the data suggesting that this phenomenon is driven by NOTCH signaling is the most compelling. Previous studies utilizing retroviral transduction of murine Lin^−^Sca-1^+^ bone marrow cells with NICD also show HSC expansion, a 30-fold expansion in CLPs, reduced differentiation capacity in methylcellulose colony-forming assays, and a block in B-cell differentiation at the CD43^+^CD19^−^ stage (Stier et al., 2002). These data, along with other studies, indicate that NOTCH1 functions at multiple levels of hematopoiesis, enhancing HSC self-renewal, promoting lymphoid-lineage commitment and T-cell versus B-cell determination (Allman et al., 2001; Pui et al., 1999; Robey et al., 1996). Transplantation of bone marrow expressing activated *NOTCH1* alleles leads exclusively to T-cell leukemia development in the mouse (Pear et al., 1996). Furthermore, NOTCH1 signaling is required for beta selection of DN thymocytes and is normally downregulated during the progression of ISP cells to the DP stage (Xiong et al., 2011). Thus, activated NOTCH1 might also explain the accumulation of ISP thymocytes and exclusive formation of TALLs in R26PR;cre animals (Fig. 7).

PRDM14 might directly regulate *Notch1* expression because it binds to *Notch1* intron 4 in mouse ESCs (Ma et al., 2011). Queries of ENCODE data suggest that this is a potential regulatory site because it is also bound by many other transcription factors and chromatin modifiers, including MYC, p300 and GCN5 (Rosenbloom et al., 2013). *Notch1* transcript, activated NICD and downstream effectors of NOTCH1 signaling, including MYC and HES1, are all expressed at high levels in R26PR-derived thymic tumors. HES1 functions as a transcriptional repressor, most notably of PTEN (a negative regulator of the PI3K-Akt signaling axis), which subsequently promotes upregulation of PI3K-Akt signaling and cellular proliferation (Palomero and Ferrando, 2008). Mouse models of PTEN loss (Tandon et al., 2011) and MYC overexpression (Smith et al., 2006) develop T-ALL with a median latency of 35 and 64 days, respectively. These survival times are remarkably similar to the R26PR;cre model, further bolstering the notion that NOTCH1 activation is a driving molecular event in R26PR;cre tumors. However, because these models are driven by genes downstream of NOTCH1, they are not as useful for the development of therapeutic strategies to target the NOTCH1 cascade in disease. Other models overexpress only NICD and/or lack the PEST regulatory domain (Murtaugh et al., 2003; Fowlkes and Robey, 2002), eliminating the ability to evaluate therapeutics targeting NOTCH1 processing, cleavage and release from the cell membrane. Our model results in *Notch1* activation from the endogenous locus, and early studies suggest that the γ-secretase inhibitor DAPT can limit proliferation of R26PR;cre T-ALL; therefore, this model will be useful for future evaluation of therapeutics targeting NOTCH1-driven human T-ALL.

In conclusion, the R26PR;cre mouse model rapidly develops TALL with complete penetrance when mated to Cre recombinases that are active in HSCs. We have previously shown that bone-marrow-transduction-derived PRDM14 leukemias are highly aneuploid, harbor numerous copy number alterations (CNAs), and have altered expression of chromosome stability, DNA repair and recombination factors (Simko et al., 2012). Thus, PRDM14-induced tumorigenesis involves not only stem cell expansion through NOTCH1 activation, but potentially induction of genomic instability. Although cancer is a multistep disease, one gene that establishes self-renewal while allowing for the accumulation of genomic aberrations could allow for the formation of CICs that produce clonal subpopulations carrying different driver mutations and characteristic genetic aberrations (Loh and Mullighan, 2012). Moving forward, the R26PR mouse line will allow for further dissection of CIC formation and progression, the development of therapeutic strategies to target CICs and modeling of additional cancer types using available Cre drivers.

## MATERIALS AND METHODS

### Animal care

All mouse experiments were carried out under the approval of the Institutional Animal Care and Use Committee at Baylor College of Medicine (BCM). Mice were housed in the Transgenic Mouse Facility (barrier level 3) under the care of the Center for Comparative Medicine, which is accredited by the Association for Assessment and Accreditation of Laboratory Animal Care International. Leukemic animals were sacrificed when moribund, and control animals at 6 or more months.

### Generation of the ROSA26 targeting construct

Mouse *Prdm14* was amplified from MIGR1-*Prdm14* (Dettman et al., 2011) using Platinum Pfx polymerase (Life Technologies, Carlsbad, CA) followed by ligation into the IRES-nls-EGFP-containing vector pBTG (Addgene plasmid 15037) using the *Nhe*I and *Xho*I restriction sites. A *Prdm14*-IRES-nls-EGFP fragment was then amplified from pBGT-*Prdm14*. This fragment was ligated into the pENTR1A dual selection vector (Life Technologies) using the *Dra*I and *Sal*I restriction sites. Gateway recombination using LR Clonase II mix (Life Technologies) was used to transfer the *Prdm14*-IRES-nls-EGFP fragment from the pENTR1A vector to pROSA26-DEST (Addgene plasmid 21189). Plasmid DNA was linearized with *Bbv*CI. Cloning primer sequences are available upon request.

### Chimeric mouse generation and breeding

Targeting vector DNA was electroporated into the C57BL/6N ESC line JM8A3 (Pettitt et al., 2009) by the Mouse Embryonic Stem Cell Core at BCM. Correctly targeted clones were microinjected into C57BL/6-*Tyr^c-Brd^* (albino) blastocysts and transplanted into pseudopregnant foster mothers by the Genetically Engineered Mouse Core at BCM. Chimeric male offspring were crossed to C57BL/6-*Tyr^c-Brd^* females to test for germline transmission of the targeted ROSA26 allele, *Gt(ROSA)26Sor^tm1Jus^* (MGI: 5502574), referred to here as R26PR. R26PR animals were crossed with the Tg(Mx1-cre)1Cgn/J line, kindly obtained from the laboratory of Dr Margaret A. Goodell (Baylor College of Medicine, Houston, TX), Tg(MMTV-cre)4Mam/J (Line D), obtained from The Jackson Laboratory, and Tg(Lck-cre)3779Nik/J (Zhang et al., 2005), obtained from The Jackson Laboratory. Mx1-cre was activated in both the R26PR;Mx1-cre and Mx1-cre lines via intraperitoneal injection of 200 μg pIpC (Sigma-Aldrich, St Louis, MO) every other day, for a total of three injections, starting at 8 weeks of age. For MMTV-cre lineage expression experiments, animals were mated to the global red/green Cre reporter line *Gt(ROSA)26Sor^tm4(ACTB-tdTomato,-EGFP)Luo^*/J (Muzumdar et al., 2007), obtained from The Jackson Laboratory.

### Southern blotting

Southern blotting was performed as previously described (Justice et al., 1994). Probes on both the 5′ and 3′ side of the insert were utilized to test accuracy of ROSA26 targeting. Probes were amplified from mouse genomic DNA via PCR with AmpliTaq Gold 360 (Life Technologies) using primers 5′-GGCTCCTCAGAGAGCCTC-3′/5′-CCGGCTGTCTCACAGAAC-3′ (5′ probe) and 5′-ACTTCCCACAGATTTTCGGTT-3′/5′-TCTCAAGCAGGAGAGTATAAAACTC-3′ (3′ probe). Genomic DNA from ESC clones was digested with *Eco*RV (WT fragment 11.5 kb, targeted fragment 4 kb) for the 5′ blot and *Pvu*II (WT fragment 5.9 kb, targeted fragment 2.3 kb) for the 3′ blot. To analyze rearrangements of the B- and T-cell receptor loci in PRDM14-induced tumors, probes to IgH, IgK, Jβ1 and Jβ2 were used as previously described (Dettman et al., 2011).

### PCR

Genomic DNA was prepared from tail biopsies for genotyping. Animals were genotyped using primers specific for the Cre transgene (5′-GCCAGCTAAACATGCTTCATC-3′/5′-ATTGCCCCTGTTTCACTATCC-3′), full-length R26PR (5′-TTCCCTCGTGATCTGCAACT-3′/5′-GCCAGAGGCCACTTGTGTAG-3′), Cre-deleted R26PR (full-length R26PR-F/5′-AGGTAGTTCGCCTTGTCCTG-3′-R), and the WT ROSA26 locus (full-length R26PR-F/5′-CCGACAAAACCGAAAATCTG-3′-R). PCR was performed using Apex Hot Start Master Mix (Genesee Scientific, San Diego, CA). For PCR analysis of TCR loci, genomic DNA from thymus, spleen and tail was amplified using Dβ2.1 and Jβ2.7 primers as previously described (King et al., 2002).

### Tissue and blood preparation

Harvested tissues were divided and snap-frozen in liquid nitrogen, and stored at −80°C for subsequent DNA/RNA extraction or were fixed in 10% neutral buffered formalin (Thermo Fisher Scientific, Waltham, MA). Formalin-fixed paraffin-embedded tissue samples were sectioned and stained with hematoxylin and eosin (Sigma-Aldrich) using standard protocols. For complete blood counts (CBCs), animals were anesthetized with isoflurane and blood was collected via the retro-orbital sinus and placed in EDTA collection tubes (Sarstedt, Nümbrecht, Germany). CBCs were performed on an Advia 120 automated hematology system. CBCs and Wright stains of blood smears were performed by the Comparative Pathology Laboratory at BCM.

### Quantitative RT-PCR

Total RNA was extracted from frozen thymi of diseased animals and controls using TRIzol Reagent (Life Technologies). RNA was reverse transcribed using the SuperScript III First-Strand Synthesis System (Life Technologies). Resulting cDNA was amplified using real-time PCR with Power SYBR Green PCR Master Mix (Life Technologies) and gene-specific primers (*Prdm14*: 5′-CCTTTGAAAAGCGCGACCGCC-3′/5′-AGGTTGAACACAGGTAGGGCCGG-3′, *TBP*: 5′-CCTTGTACCCTTCACCAATGAC-3′/5′-ACAGCCAAGATTCACGGTAGA-3′, *Notch1*: 5′-GGATGTCAATGTTCGAGGA-3′/5′-CAGCAGGTGCATCTTCTTCT-3′). Amplification and data analysis were conducted on a Rotor-Gene Q machine and software (Qiagen, Valencia, CA). Relative gene expression was calculated using the ΔΔC_T_ method (Pfaffl, 2001).

### Flow cytometry

Flow cytometry was performed on peripheral blood, total bone marrow, thymus or spleen. Bone marrow cells were isolated by crushing bones with a mortar and pestle wet with Hank’s Balanced Salt Solution (Life Technologies) and filtering through a 0.45-μm cell strainer (BD, Franklin Lakes, NJ). Peripheral blood was prepared by layering blood obtained from the retro-orbital sinus onto a 1% dextran/heparin solution and allowing it to settle. The upper phase was removed and used for subsequent analysis. Thymus and spleen tissue was minced with a razor and filtered through a cell strainer. Cells were stained with the following fluorophore-conjugated antibodies from eBioscience (San Diego, CA) [B220-PE-Cy7 (25-0452), CD4-Alexa Fluor 700 (56-0041), CD4-PE (12-0041), CD8-eFluor 450 (48-0081), CD24-PE (12-0241), CD25-APC (17-0251), CD34-Alexa Fluor 700 (56-0341), CD43-PE (12-0431), CD44-PECy7 (25-0441), CD150-PE (12-1502), c-Kit-APC-eFluor 710 (47-1171), Flt3-PE (12-1351), IgM-APC (17-5790), IL-7Rα-eFluor 450 (48-1271), Mac1-PE (12-0112), Sca1-APC (17-5981)] or BD Pharmingen (San Diego, CA) [B220-Pacific Blue (558108), B220-PE (553090), CD3-PE-Cy7 (552774), CD16/32-PE (553145), CD19-PE-Cy7 (552854), Lineage Cocktail-APC (558074) and PerCP-Cy5.5 (561317), TCRβ-APC (561080)]. Samples were subsequently analyzed on a LSR Fortessa flow cytometer (BD) in the Flow Cytometry and Cell Sorting Core at BCM. Data interpretation was conducted using FACSDiva (BD) and FlowJo (TreeStar Inc., Ashland, OR) software.

### MethoCult assay

R26PR;Mx1-cre and Mx1-cre animals were injected with pIpC and bone marrow was harvested 2 weeks later. Bone marrow was sorted for Lineage^−^ Sca-1^+^ c-Kit^+^ CD150^+^ cells and was seeded at a density of 500 cells per replicate in methylcellulose supplemented with myeloid growth factors including SCF, IL-3, IL-6 and Epo in 35-mm culture dishes with grids (MethoCult, M3434, STEMCELL Technologies, Vancouver, BC, Canada). Total colony numbers, including BFU-E, CFU-GM and CFU-GEMM, were counted at day 12 in culture.

### Western blot analysis

Protein lysates were prepared from frozen thymi of diseased animals and controls using T-PER reagent (Thermo Scientific, Waltham, MA) supplemented with protease inhibitors (Roche, Indianapolis, IN). 30 μg of lysate were separated on a 4–20% gradient polyacrylamide gel (Bio-Rad, Hercules, CA) and blotting was performed using standard protocols. Primary antibodies used were from Cell Signaling Technology (Danvers, MA) [cleaved NOTCH1 (4147S)], EMD Millipore (Billerica, MA) [MYC (06-340) and HES1 (AB5702)] and Sigma-Aldrich [ACTIN (A2066)]. Secondary HRP-goat-anti-rabbit antibody was obtained from Jackson ImmunoResearch (West Grove, PA).

### Tumor cell culture

Tumor cell lines were derived from primary R26PR;MMTV-cre thymic tumors by mincing and passing through a cell strainer. Cells were cultured in RPMI-1640 (Lonza, Basel, Switzerland) supplemented with L-glutamine, penicillin/streptomycin, 10% FBS and 50 μM β-mercaptoethanol. For DAPT treatment, cells were seeded at 2×10^5^ cells/ml in T25 flasks in media supplemented with 1 μM DAPT (Selleck Chemicals, Houston, TX) or 0.1% DMSO vehicle. Live cells were counted every 24 hours using trypan blue exclusion and a TC10 cell counter (Bio-Rad). Cell culture experiments were performed in triplicate.

### Data analysis

Graphical and statistical analyses were conducted using Prism (GraphPad, La Jolla, CA). The log-rank test was performed for survival analysis, and a one-way ANOVA with Dunnett’s correction for multiple comparisons was performed on data with three or more groups. All other analyses were performed using *t*-tests.

## Supplementary Material

Supplementary Material
