# Supplementary material for: A mouse model for inducible overexpression of Prdm14 results in rapid-onset and highly penetrant T-cell acute lymphoblastic leukemia (T-ALL)
Source: Dis Model Mech. 2013 Sep 5;6(6):1494–506. doi: 10.1242/dmm.012575 (PMC3820272; doi:10.1242/dmm.012575)
Supplement: Supplementary Material [file supp_6_6_1494__index.html]

A mouse model for inducible overexpression of Prdm14 results in rapid-onset and highly penetrant T-cell acute lymphoblastic leukemia (T-ALL) — A mouse model for inducible overexpression of Prdm14 results in rapid-onset and highly penetrant T-cell acute lymphoblastic leukemia (T-ALL) — Supplementary Material 

# A mouse model for inducible overexpression of *Prdm14* results in rapid-onset and highly penetrant T-cell acute lymphoblastic leukemia (T-ALL)

## DMM012575 Supplementary Material

**Files in this Data Supplement:**

- **Supplementary Material PDF**
