# Supplementary material for: A mouse model for inducible overexpression of Prdm14 results in rapid-onset and highly penetrant T-cell acute lymphoblastic leukemia (T-ALL)
Source: Dis Model Mech. 2013 Sep 5;6(6):1494–506. doi: 10.1242/dmm.012575 (PMC3820272; doi:10.1242/dmm.012575)
Supplement: Supplementary Material [file supp_012575_DMM012575.pdf]

SUPPLEMENTARY MATERIAL

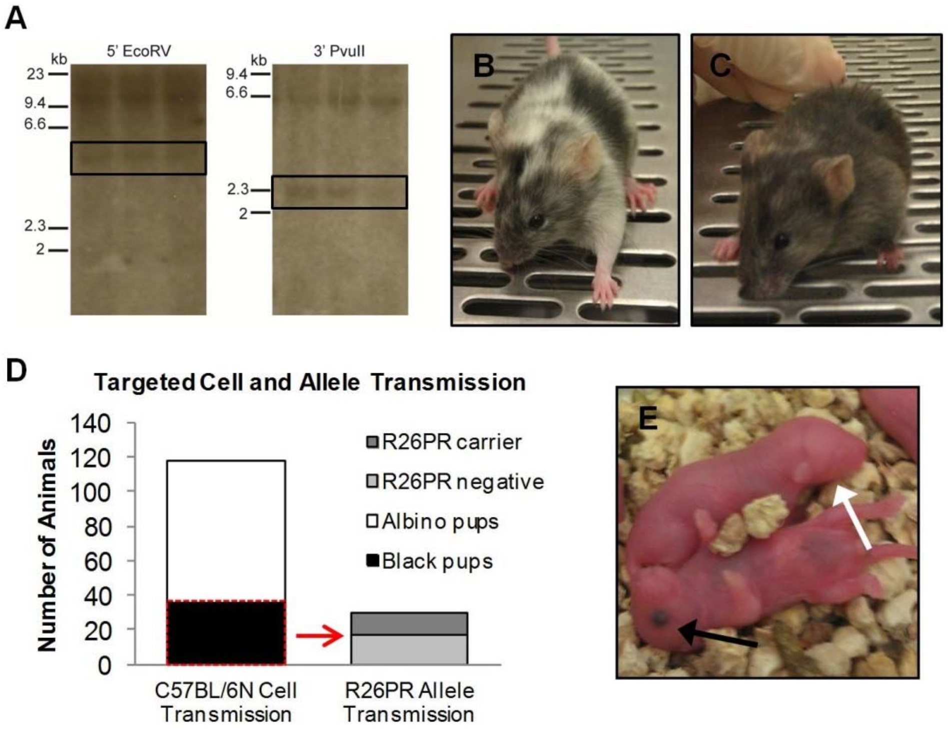

Fig. S1. Generation of the R26PR mouse line. (A) Southern blot analysis to confirm appropriate targeting of the FS-*Prdm14* construct to the ROSA26 locus. A probe falling of the 5' side of the ROSA26 locus was used following digestion of genomic DNA from targeted embryonic stem (ES) cells with EcoRV. A 3' probe was used following digestion of genomic DNA with PvuII. The black box indicates the correctly targeted band, which was present in the three clones shown. The three clones shown were used for subsequent blastocyst injections. (B,C) Representative chimeras derived from blastocyst injections. Dark coat color is derived from the targeted ES cells. Animals of high coat color contribution were mated for germline transmission. (D) Germline transmission was first detected visually by identifying animals with dark coats and eyes, indicating they were derived from the targeted ES cells. Dark pups were then genotyped for the presence of the targeted R26PR allele. 35 dark-coated animals were genotyped and 13 were carriers of the R26PR allele. (E) Representative image of dark (derived from targeted ES cells) and albino (derived from host blastocyst) pups from a germline transmission mating. Black arrow indicates dark eyes, white arrow indicates albino eyes.

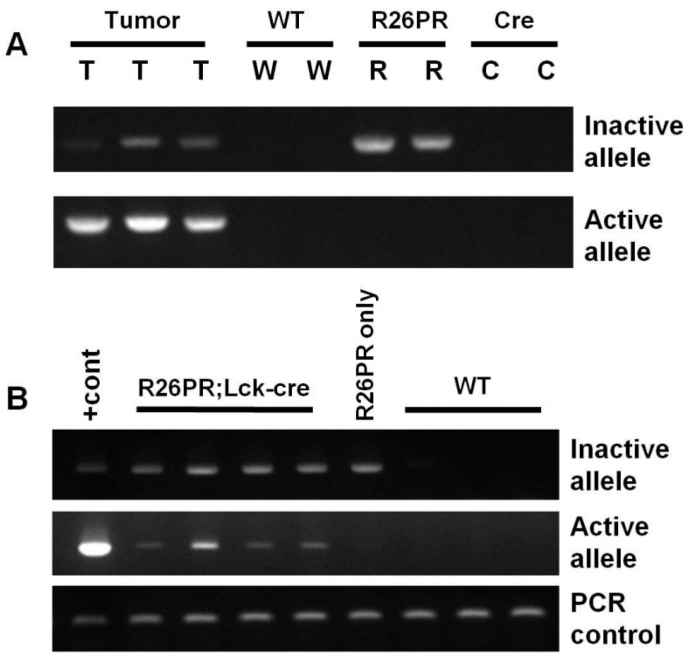

Fig. S2. PCR analysis of genomic DNA to determine status of the floxed stop cassette. The "inactive allele" PCR will specifically detect the R26PR allele only when the FS cassette is still present. The "active allele" PCR will detect the R26PR allele only following Cre-mediated excision of the FS cassette. (A) Analysis of allele status in R26PR;MMTV-cre tumors and controls. The active allele was detected in tum or tissue from the R26PR;MMTV-cre line (lower panel). Additionally, residual inactive allele was present in tumors and in R26PR-only controls (upper panel). (B) Analysis of allele status in R26PR;dLck-cre thymus and control thymus. The active allele was detected at low levels in all R26PR;dLck-cre animals (middle panel), indicating Cre was functional in this model. R26PR;dLck-cre and R26PR-only controls also contained the inactive allele (upper panel). An internal PCR control is also shown (lower panel).

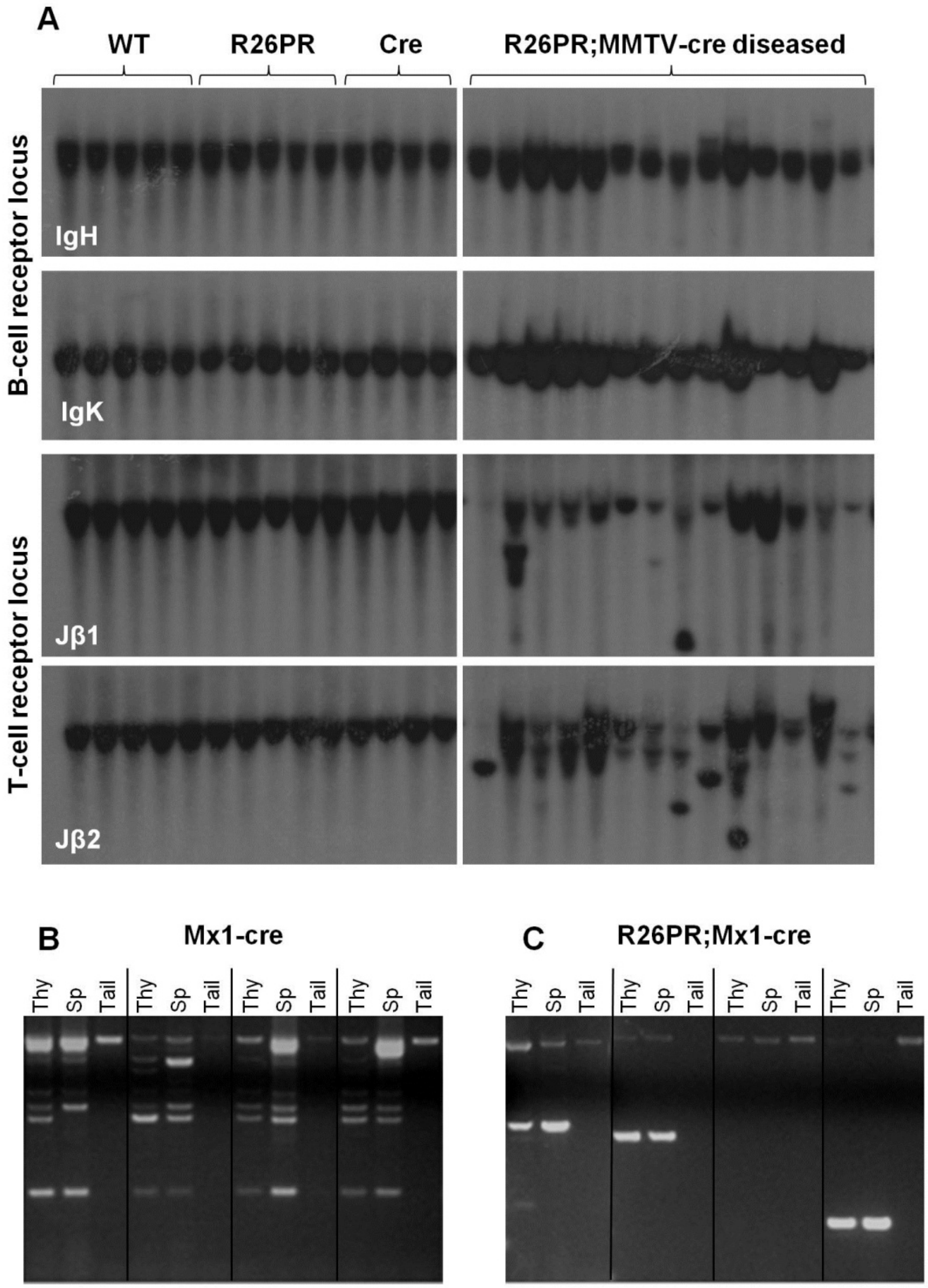

Fig. S3. Analysis of B-cell receptor (BCR) and T-cell receptor (TCR) loci configuration in tumors and control tissue. Rearrangement of BCR and TCR can be used to determine the cell of origin within a lymphoid tumor (B- or T-cell) and indicate the clonality of the tumor. (A) Southern blot analysis of R26PR;MMTV-cre spleens and control spleens. No dominant BCR rearrangements (immunoglobulin heavy [IgH] or light [IgK] chains, upper two panels) were present in any of the samples and the primary band observed was for the germline BCR configuration. No TCR rearrangements were present in any of the controls (Jβ1 and J β2, lower two panels), but rearrangements were detected in tumor tissue, indicating leukemia was T-cell in origin. Some samples had multiple dominant clones, indicating oligoclonal disease development in those animals. (B,C) PCR analysis of TCR using primers to Dβ2.1 and Jβ2.7 using gDNA from thymus (Thy), spleen (Sp), and tail tissue in Mx1-cre animals (B) and R26PR;Mx1-cre animals (C). Tail tissue always contained the germline TCR configuration. Dominant clones of the same size were detected in both thymus and spleen of affected animals, indicating that peripheral disease was derived from the original tumor.
